# Can Ivermectin kill *Sarcoptes scabiei* during the molting process?

**DOI:** 10.1371/journal.pntd.0011337

**Published:** 2023-05-17

**Authors:** Shenrui Feng, Minmin Shi, Zhijuan Yin, Wenda Di, Jacques Guillot, Fang Fang

**Affiliations:** 1 Parasitology Department, College of Animal Science and Technology, Guangxi University, Nanning, China; 2 Dermatology-Parasitology-Mycology Departement, ONIRIS, Nantes, France; Federation University Australia, AUSTRALIA

## Abstract

**Background:**

*Sarcoptes scabiei* is a permanent obligate ectoparasite that lives and reproduces in the epidermis of humans and other mammals worldwide. There is a lack of information on the molting process of *Sarcoptes scabiei*. Ivermectin is widely used to treat *Sarcoptes* infection in humans and animals, while the survival of molting *Sarcoptes* mites in the presence of ivermectin is unknown. The aim of the present study is to investigate the molting process of *Sarcoptes* mites and assess the activity of ivermectin during the molting process of *Sarcoptes* mites.

**Methodology/Principal findings:**

molting *Sarcoptes* mites were incubated at 35°C and 80% relative humidity and observed hourly until complete molt. Of the 192 molting mites recorded, the longest molt periods for larvae and nymphs were 23 and 30 h, respectively. The activity of ivermectin on molting *Sarcoptes* mites was also assessed using two concentrations of the drug (0.1 and 0.05 mg/ml). The exposure time for molting mites was determined by 100% mortality of female mites exposed to the solution of ivermectin. While all female mites were killed after exposure to 0.1 mg/ml ivermectin for 2 h and and 0.05 mg/ml for 7 h, 32% and 36% of molting mites survived and successfully molted, respectively.

**Conclusions/significance:**

The present study demonstrated that molting *Sarcoptes* mites are less susceptible to ivermectin than active mites. As a consequence, mites may survive after two doses of ivermectin given 7 days apart due not only to hatching eggs but also to the resistance of mites during their molting process. Our results provide insight into the optimal therapeutic regimens for scabies and highlight the need for further research on the molting process of *Sarcoptes* mites.

## Author summary

*Sarcoptes scabiei* is a permanent obligate ectoparasite causing contagious disease in humans and other mammals worldwide. *Sarcoptes* mites undergo three molts in their lifetime, however, there is limited information on their molting process. Oral ivermectin is widely used for the treatment of *Sarcoptes* infection in humans and animals. To investigate whether ivermectin is active against molting *Sarcoptes* mites, we first explored the molting process of *Sarcoptes* mites *in vitro*, and then assess the activity of ivermectin. We demonstrated that the average molt period lasts for about 2 days. When exposed to ivermectin, one-third of molting mites survived whereas all active mites died. The present study suggests that mites may survive after two doses of ivermectin 7 days apart due to the resistance of mites during the molting process.

## Introduction

*Sarcoptes scabiei* is a permanent obligate ectoparasite that lives and reproduces in the epidermis of humans and other mammals worldwide [1]. The life cycle of *Sarcoptes scabiei* includes eggs, larvae, protonymphs, tritonymphs and adult males or females [2]. During their development, *Sarcoptes* mites undergo three molts: from larvae to protonymphs, from protonymphs to tritonymphs and from tritonymphs to adults. The molting process of *Sarcoptes scabiei* is still unknown and there is no information about the susceptibility of molting *Sarcoptes* mites to acaricides [3].

Ivermectin is commonly used for the treatment of *Sarcoptes* infection in both humans [4,5] and animals [6]. Studies have demonstrated its effectiveness against various mite species, including *Psoroptes cuniculi* [7], *Cheyletiella* mites [8], *Chorioptes* mites [9] and *Demodex* mites [10]. Ivermectin has been shown to be active against the motile stages of *Sarcoptes scabiei*, with *in vitro* tests revealing an LC_50_ of 1.8 μM at 24h. However, ivermectin is not effective against all stages of *Sarcoptes* mites [11], and suboptimal responses in some cases [12,13] indicate a need for more detailed investigation in mite biology. A study proved that the eggs of *Sarcoptes* mites cannot be effectively killed by Ivermectin [14].

In the present study, we collected molting *Sarcoptes* mites to explore their molting process and to assess the activity of two concentrations of ivermectin.

## Methods

### Ethics statement

Mites were obtained from naturally infected New Zealand white rabbits in a rabbit farm in Nanning, Guangxi Province, China, with ethical approval from the ethics committee of Guangxi University (approval no: GXU2019-019).

### *Sarcoptes* mites and compounds

**Fig 1:**
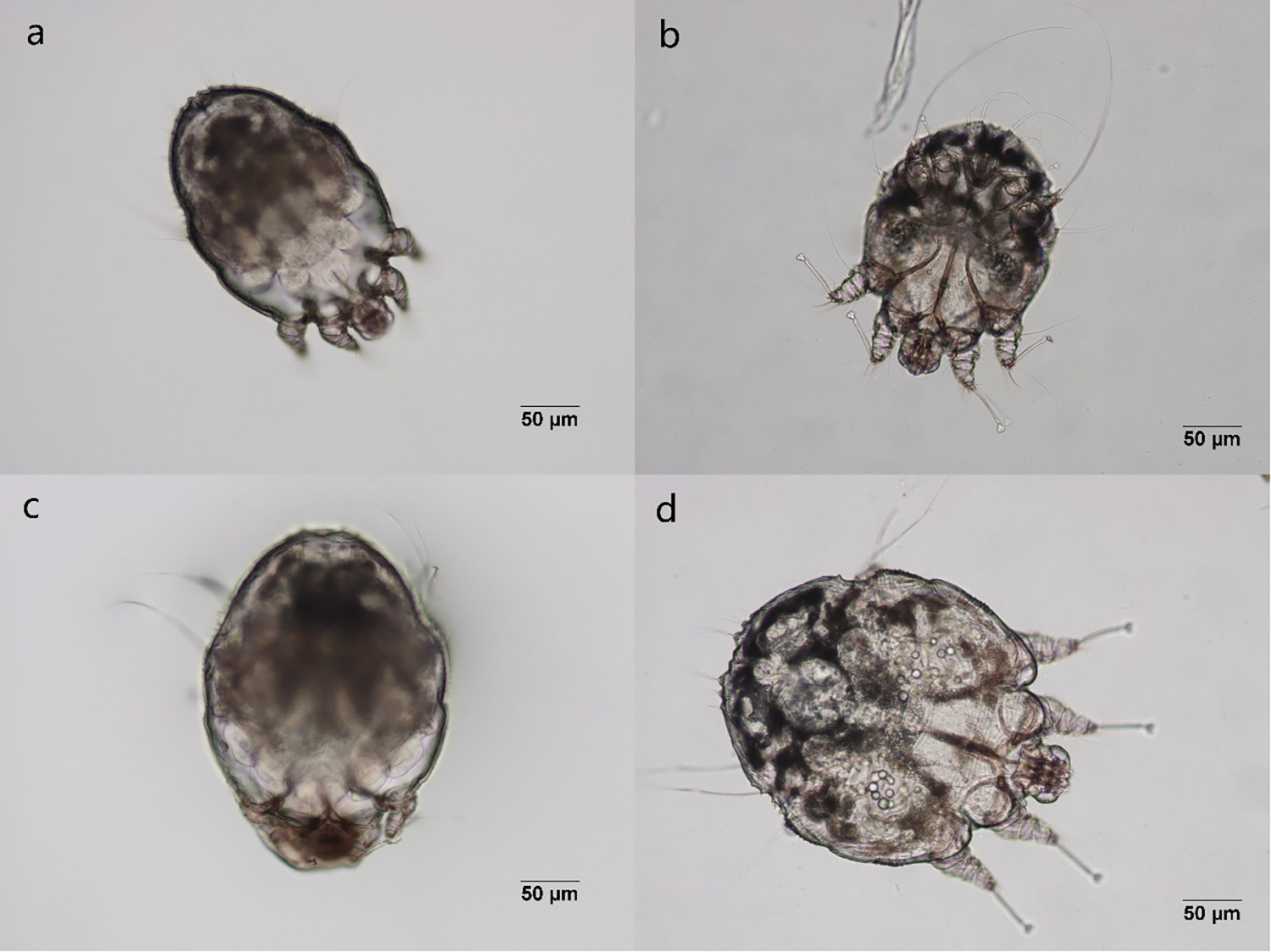
The molting process of *Sarcoptes scabiei* (200x). a: a molting male mite; b: the molted male mite; c: a molting female mite; d: the molted female mite.

Heavily-infected crusts were placed in Petri dishes and transported to the laboratory within a half-hour. *Sarcoptes* mites were isolated one by one with a needle under a stereomicroscope (SMZ745; Nikon Corp., Tokyo, Japan; 2xmagnification). Mites that were covered with an outer cuticle and remained motionless were considered as molting mites (Fig 1).

Ivermectin (≥95%) was purchased from Beijing Solarbio Science & Technology Co., Ltd. Paraffin was purchased from Shanghai Macklin biochemical company, Shanghai, China. We used 10% Medium-chain triglycerides (Medium-chain triglycerides∶Paraffin = 1∶9) as a solvent for ivermectin, which was purchased from the Nisshin OilliO Group, Ltd.

### Molting process of *Sarcoptes* mites

Molting *Sarcoptes* mites were placed on glass slides. All slides were incubated at 35°C and 80% relative humidity. The status of molting mites was examined under a stereomicroscope (Eclipse 80i; Nikon Corp.; 20×magnification) at hourly intervals until molt.

### Assessment of ivermectin activity

To test the efficacy of ivermectin on molting *Sarcoptes* mites, we first exposed female mites to 0.1 and 0.05mg/ml ivermectin diluted in 10% Medium-chain triglycerides (MTC). Acaricidal sensitivity assays were performed using methods previously described [15]. To be brief, female mites (n = 10) were exposed to each concentration of ivermectin with five replicates. In each Petri dish, 1 ml of the solution was included in direct contact with the mites. A control Petri dish was inoculated with 1 ml of 10% MTC. Mites were considered dead when no movement was seen even after touching them with a needle. The exposure time for molting mites was determined by 100% mortality of female mites exposed to the solution of ivermectin. Molting mites (n = 10) were then exposed to 0.1 and 0.05mg/ml ivermectin, respectively. Molting mites were washed and incubated in a humidity chamber (35°C, 80% relative humidity) for the subsequent molting process. Five replicates were performed for each concentration. Negative controls (n = 10) consisted of molting mites exposed to 10% MTC. Mortality and the status of molting mites were measured after 48 h. Five replicates were performed for each concentration of ivermectin. The molting *Sarcoptes* mites were considered dead if they failed to molt after 48h since no more molting *Sarcoptes* mites molt after this time point.

### Data analysis

Data were analyzed by SPSS software version 23.0. The molting time of *Sarcoptes* mites was analyzed with mean ± standard deviation. Survival of molting *Sarcoptes* mites and female mites exposed to ivermectin was compared by one-way analysis of variance (ANOVA) followed by LSD (Least Significant Difference). Values of p < 0.05 were considered significant. The median Lethal Concentration (LC_50_) was calculated by probit regression analysis. Columns were plotted by Graphpad Prism 9.0 software.

## Results

**Fig 2:**
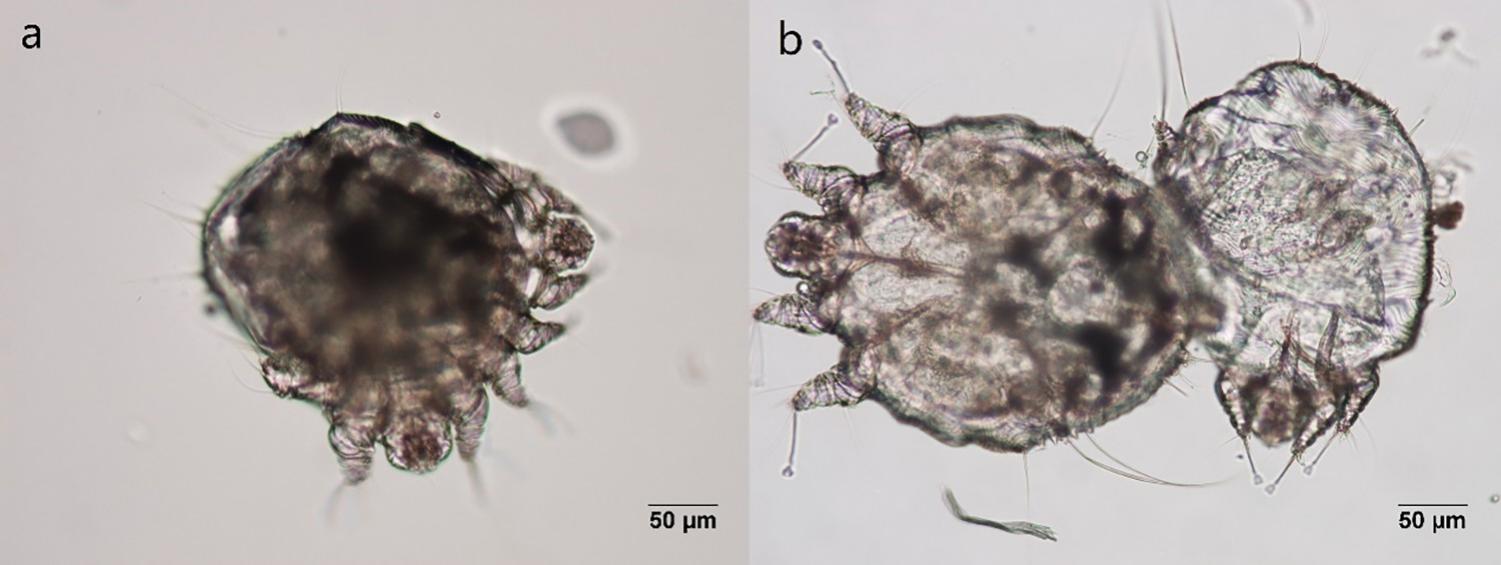
a: a mite bites through the old cuticle. b: a mite goes out from the old cuticle (200x).

**Fig 3:**
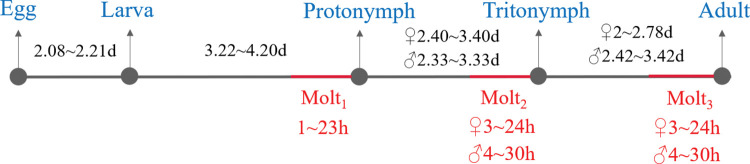
The life cycle of *Sarcoptes scabiei* (adapted from Arlian and Vyszenski-Moher [2]).

To determine the duration of the molting period, 192 molting *Sarcoptes* mites were collected and examined. Mites during the molting process were quiescent (Fig 1). When they start to molt, they bite through the old cuticle using capitulum (Fig 2). Of the 192 molting mites recorded, the average duration of the molting period was 12.6 ± 5.9 h for 51 larvae/protonymphs, 17.8 ± 6.9h for 32 males (including protonymphs/tritonymphs and tritonymphs/males), and 15.1 ± 5.7h for 106 females (including protonymphs/tritonymphs and tritonymphs/females). The longest molting periods observed were 23, 30, and 24 h for protonymphs, males (including protonymphs/tritonymphs and tritonymphs/males) and females (including protonymphs/tritonymphs and tritonymphs/females), respectively (Fig 3). Three molting mites died during the incubation.

**Fig 4:**
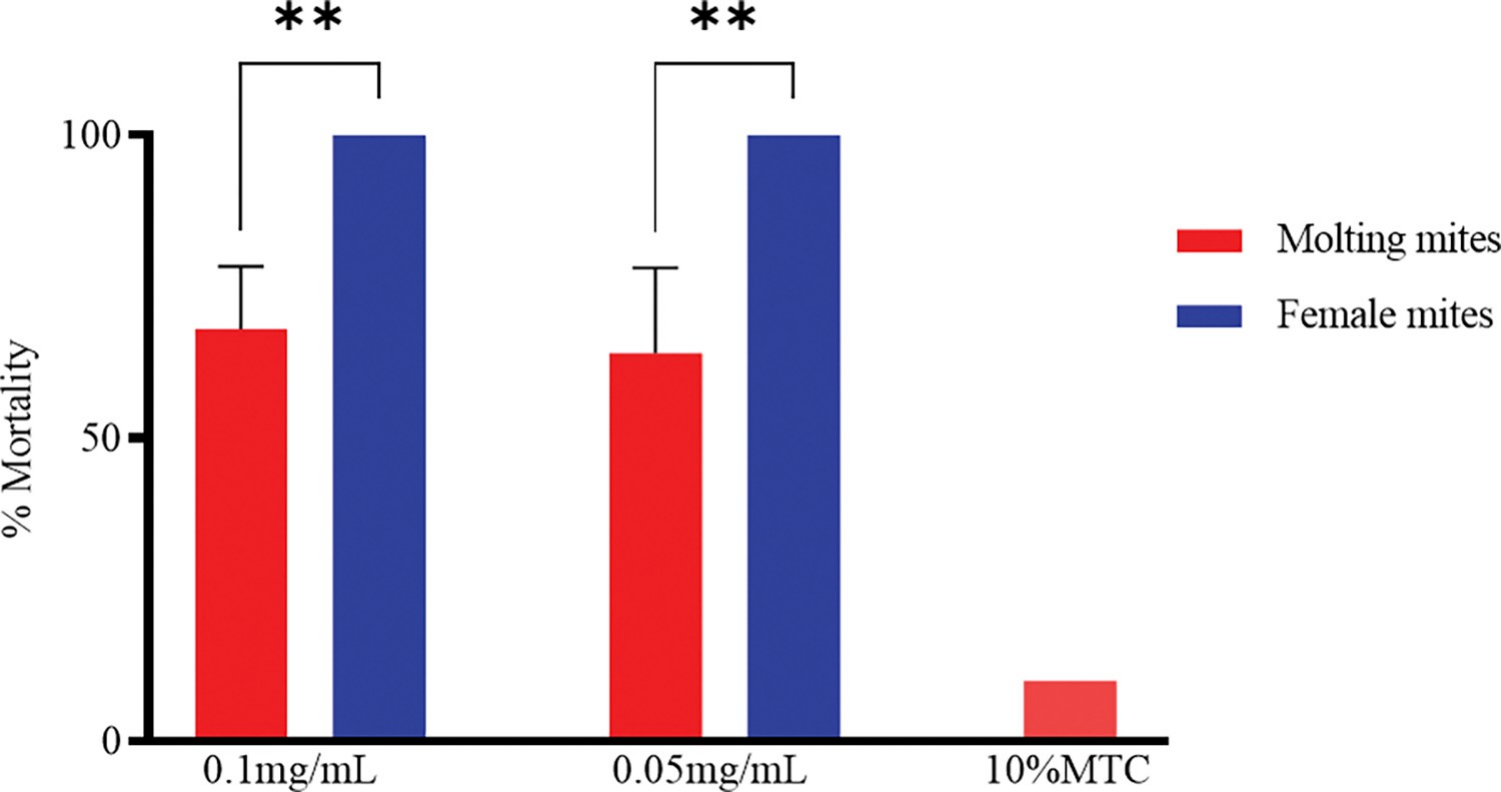
The mortality of molting *Sarcoptes* mites and females exposed to 0.1 mg/mL (2h) and 0.05 mg/mL (7h). The control is molting *Sarcoptes* mites exposed to 10%MTC. **: P<0.01.

When exposed to 0.1 and 0.05 mg/ml of ivermectin, all female mites were dead in 2 and 7 h, respectively. After 2 h exposure to 0.1mg/ml ivermectin, 32 ± 10.4% of mites were still able to molt successfully. After 7 h of exposure to 0.05 mg/ml ivermectin, 36 ± 14.2% of mites were still able to molt successfully (Fig 4). Significant differences (p<0.01) were observed between the survival rates of females and molting mites exposed to 0.1 mg/ml and 0.05 mg/ml of ivermectin for 2 and 7 h. No significant difference (p>0.05) were observed between the molting *Sarcoptes* mites exposed to 0.1 mg/ml and 0.05mg/ml ivermectin for 2 and 7 h.

## Discussion

The present investigation demonstrated for the first time how long it takes for *Sarcoptes* mites to molt. During the 10–13 day-duration of the life cycle of *Sarcoptes* mites [2,16], the average molt period could last for 2 days. Since it is not possible to incubate the molting mites from the start of the molting process, the duration of one molt period could last over 30 h.

Our findings suggest that molting mites are less susceptible to ivermectin than motile mites. This might be attributed to the outer cuticle which offers protection to the mites [16,17]. In the present study, the LC_50_ for ivermectin against female mites at 1h was 84.1 μM, which is higher than the values reported in previous studies (50.5 μM and 45.1 μM) [18,19]. This could be due to mite resistance since the rabbits had been treated with ivermectin before the investigation.

The molt stage is a crucial point in the life cycle of arthropods, during which the old exoskeleton is shed, and a new one is formed. Currently, there is limited information available on the molting process of parasitic mites. Our findings may enhance the understanding of the life cycle of other parasitic mites.

Studying the molting process of mites could lead to the development of new drugs that target the molt stage. A class of molecules known as benzoylphenyl ureas (BPUs) can disrupt chitin synthesis in arthropod. This disruption prevents the formation of the exoskeleton necessary for molting, ultimately leading mites to death [20]. BPUs, such as diflubenzuron and flufenoxuron, have been found to be effective against several species of mites, including *Tetranychus urticae* [21] and *Panonychus citri* [22]. Juvenile hormone analogs mimic the effects of juvenile hormone and can disrupt the normal molting process in mites, preventing them from maturing [23]. Methoprene and fenoxycarb have been found to be effective against house dust mite (*Dermatophagoides farina*) [24,25]. These drugs offer a notable benefit as they target arthropod-specific physiological and biochemical mechanisms that are not present in vertebrates, thus rendering them practically non-toxic to mammals [26].

Oral ivermectin has become the first-line treatment option for both classical and crusted scabies [27]. It is the only systemic treatment currently available, at a dose of 150–200 μg/kg, to be repeated after 7 days [28]. After oral administration, the maximum concentration (*C*_*max*_) in plasma of ivermectin in humans was 20.2–81.0 ng/ml [29]. A study of pigs measured the *C*_*max*_ of ivermectin in the skin as 54.8 ng/ml and the *t*_1/2_ as one day [30]. Concerning the short half-life of ivermectin, mites during the molting process may survive. For example, if an infested individual receives oral ivermectin, a larva during the molting process may remain on the individual and molt into a protonymph. This protonymph could survive a second dose of ivermectin after 7 days, since it may have progressed to the tritonymph- adult molt stage and eventually develop into an adult, thus continuing the cycle of infection. This may explain why the pigs under heavy infection couldn’t be cured completely after two doses of ivermectin, while hatched egg should have been killed by the second treatment [30,31]. Therefore, to clear the infection successfully, a second treatment may be required after the mites have completed their molting process.

One limitation of the current study is that only two concentrations of ivermectin were tested. Collecting molting mites in large quantities is extremely difficult since they remain quiescent, typically located deep within the crust, leading them complicated to work with and even more difficult than mites’ eggs.

The molting process of *Sarcoptes* mites has been disregarded due to the challenges associated with finding molting mites and the absence of an in vitro culture system. Our discovery of the molt stage offers promising directions for future research. Additional concentrations of ivermectin must be tested to determine the concentration required to kill 50% of molting mites. The efficacy of other acaricides, such as permethrin or moxidectin, should be evaluated against molting *Sarcoptes* mites. Furthermore, it is worth investigating molecules that have been proven to target the molt stage of arthropods to gain a better understanding of their potential for controlling parasitic mite infestations.

In conclusion, this work provided complementary and original information regarding the life cycle of *Sarcoptes scabiei*. It clearly demonstrated that molting *Sarcoptes* mites are less susceptible to ivermectin than motile mites. This finding could be useful in refining the treatment approach for scabies.

## Supporting information

S1 Video A mite about to molt. (MP4)

S1 Table Raw experimental data. (XLSX)
